# Are changes in patient-reported outcomes prognostic for diffuse large B-Cell lymphoma survival? Results from the GOYA trial

**DOI:** 10.1186/s41687-024-00708-w

**Published:** 2024-03-18

**Authors:** Jessie T. Yan, Célia Bel, Peter C. Trask, Ernest Lo

**Affiliations:** 1 Roche Information Solutions, Roche Diagnostics, 2881 Scott Blvd, 95050 Santa Clara, CA USA; 2 Diagnostics Division, F. Hoffmann-La Roche Ltd, Basel, Switzerland; 3 Genentech Inc, South San Francisco, CA USA

**Keywords:** B-cell lymphoma symptoms, Diffuse large B-cell lymphoma, Patient-reported outcomes, Patient-reported outcome measures, Survival

## Abstract

In this hypothesis-generating analysis, we examined whether longitudinal changes in patient-reported outcomes (PROs), such as symptoms, over time would be prognostic for progression-free survival (PFS) and overall survival (OS) in patients with diffuse large B-cell lymphoma (DLBCL) who were newly treated with obinutuzumab (G) in combination with CHOP (G-CHOP) or rituximab (R) with CHOP (R-CHOP), in the GOYA Phase 3 trial (NCT01287741). Our results show that from the study baseline to cycle 3 day 1, every 1-point increase (worsening) in fever symptoms was associated with a 41% higher risk of death (hazard ratio [HR], 1.41; *P* = 0.01). Every 1-point increase (worsening) in lumps or swelling symptoms was associated with a 27% higher risk of disease progression or death (PFS events) (HR, 1.27; *P* = 0.01) and a 29% higher risk of death (OS events) (HR, 1.29; *P* = 0.02). No significant associations were observed between survival and changes in other symptoms, such as itching. Our study suggests that changes in some PROs are related to survival in DLBCL patients.

**Supplementary Information:**

The online version contains supplementary material available at 10.1186/s41687-024-00708-w.

## Introduction

Diffuse large B-cell lymphoma (DLBCL) is the most common type of aggressive non-Hodgkin lymphoma [1]. Despite the availability of first-line therapy of rituximab (R) and an anthracycline-containing regimen, such as CHOP (cyclophosphamide, doxorubicin hydrochloride [Adriamycin], vincristine sulfate [Oncovin], and prednisone), more than one-third of patients do not respond or become refractory to first-line therapy and die within 5 years of diagnosis [2].

To improve health outcomes in DLBCL patients, it would be useful to understand those clinical factors that have prognostic significance. Evidence from nascent literature suggests that when assessed at a single time point, patient-reported outcomes (PROs), including those measuring health-related quality of life (HRQOL) and symptoms, can be prognostic for progression-free survival (PFS) and overall survival (OS) in patients with cancer [3]. A recent analysis of 1,239 patients with DLBCL from the GOYA trial found that baseline PROs provided prognostic information for both PFS and OS [4]. However, little is known about how longitudinal changes in PROs may impact treatment outcomes, particularly in patients with DLBCL. In practice, clinicians often use changes in a patient’s cancer-related symptoms to evaluate treatment response and inform prognosis.

Building upon the previous work [4], the objective of this analysis was to examine the association between changes in PROs over time and PFS and OS in patients with DLBCL who were newly treated with obinutuzumab (G) in combination with CHOP (G-CHOP) or rituximab (R) with CHOP (R-CHOP), in the GOYA Phase 3 trial.

## Methods

### Study design

The GOYA trial was a Phase 3, multicenter, open-label, randomized trial comparing the efficacy of G-CHOP and R-CHOP in previously untreated patients with CD20-positive DLBCL [5]. The study design of the GOYA trial (NCT01287741) has been previously reported [5]. Final analysis of data from this trial showed no significant difference in PFS or OS in the G-CHOP or R-CHOP treatment groups [5].

### Patient-reported outcome measures

The GOYA trial included 2 patient-reported outcomes measures (PROMs), the European Organization for Research and Treatment of Cancer Quality of Life Questionnaire (EORTC QLQ-C30) and the Functional Assessment of Cancer Therapy-Lymphoma (FACT-Lym) Lymphoma Subscale (LymS).

The EORTC QLQ-C30 is a commonly used, validated, and reliable instrument for measuring HRQOL in people with cancer [6]. The instrument includes 30 items that are divided into a global health status/quality of life scale, 5 functional scales (physical, role, cognitive, emotional, and social), 8 symptom scales (fatigue, pain, nausea and vomiting, dyspnea, insomnia, appetite loss, constipation, and diarrhea), and a financial consequences scale [7]. The recall period is one week. Most items use a 4-point scale (1 = not at all, 2 = a little, 3 = quite a bit, and 4 = very much). The scores are converted to scale scores ranging from 0 to 100 points according to the EORTC scoring manual [7]. A higher global health or function domain score indicates better function while lower scores in the symptom scales indicate less symptom severity.

The FACT-Lym LymS is a 15-item subscale of the 42-item FACT-Lym questionnaire and has been shown to demonstrate reliability and validity in patients with non-Hodgkin lymphoma [8]. The questions consist of common lymphoma disease or treatment-related symptoms (pain, lumps or swelling, fever, night sweats, trouble sleeping, itching, weight loss, fatigue, and loss of appetite) and potential illness-related concerns (e.g., feeling isolated from others and emotional ups and downs) [9]. The recall period is the past 7 days. Each item is scored on a 5-point scale (0 = not at all, 1 = a little bit, 2 = somewhat; 3 = quite a bit; 4 = very much) [9]. The FACT-Lym LymS score ranges from 0 to 60 points. A higher score relates to a better HRQOL.

Patients from the GOYA trial were included in this secondary analysis if they completed the EORTC QLQ-C30 and FACT-Lym LymS at both study baseline and Cycle 3 Day 1 (C3D1). Completion of PROMs was defined as completion of ≥ 50% of the items per subscale. For the EORTC QLQ-C30, a change in score of 10 points was considered the minimal important difference (MID) [10] or the smallest amount of change considered important to patients. For the FACT-Lym LymS, a change in score of 3 points was used to define the proportion of patients reporting a MID [11]. For individual items under the FACT-Lym LymS, a cutoff for MID is not yet well defined. Based on author consensus and a review of the literature, a change in score of 1 point was used to define a MID for the individual items of the FACT-Lym LymS, with higher score indicating worsening symptoms. The approach of detecting a MID based on a single item has been used in other studies [12, 13].

### Statistical analysis

We analyzed the changes in PROMs from baseline to C3D1, a midway checkpoint for a typical treatment course for patients with DLBCL (typically lasting 6–8 cycles in total). Changes in PROM scores were treated as continuous variables.

To assess the associations between changes in PROMs and survival (i.e., both PFS and OS), Cox regression models were performed with the hazard ratios (HRs) rescaled to represent a MID. As the survival outcomes did not differ by treatments (G-CHOP vs. R‐ CHOP) in GOYA [5], similar to the previous work [4], we combined data from both treatment arms to increase statistical power (i.e., more events for PFS and OS analyses). However, as a precaution all Cox regression analyses were stratified by treatment arms.All models were further adjusted for disease risk (lower vs. higher) according to the International Prognostic Index (IPI) score (low/low-intermediate, 0–2; high/high-intermediate, 3–5) at diagnosis, cell of origin, *BCL2* status, total metabolic tumor volume, and baseline PRO score.

No multiple testing was performed in this hypothesis-generating analysis. Statistical analyses were performed using R software version 3.6.3.

## Results

Our study included 1,132 patients with the mean age of 58.5 years (standard deviation [SD], 13.6) **(**Table 1**)**.

**Table 1:** Baseline Characteristics of Patients From the GOYA Trial Who Completed the EORTC QLQ-C30 and FACT-Lym LymS at Both Baseline and Cycle 3 Day 1

Characteristic	*N* = 1,132
Age, y	
Mean (SD)	58.5 (13.6)
Median [Min, Max]	61.0 [18.0, 86.0]
TMTV, cm^3^	
Mean (SD)	662 (968)
Median [Min, Max]	337 [1.04, 10 600]
Missing	80 (7.1%)
Sex	
Female	529 (46.7%)
Male	603 (53.3%)
Bone marrow involvement	
Positive	118 (10.4%)
Negative	994 (87.8%)
Indeterminate	11 (1.0%)
Missing	9 (0.8%)
*BCL2* mutational status	
Positive	282 (24.9%)
Negative	313 (27.7%)
Missing	537 (47.4%)
Cell of origin	
ABC	196 (17.3%)
GCB	428 (37.8%)
Unclassified	117 (10.3%)
Missing	391 (34.5%)
ECOG PS	
0–1	1004 (88.7%)
2–3	127 (11.2%)
Missing	1 (0.1%)
Ann Arbor Stage	
I-II	277 (24.5%)
III-IV	855 (75.5%)
Missing	0 (0%)
LDH, units/L	
≤ 280	565 (49.9%)
> 280	564 (49.8%)
Missing	3 (0.3%)
Extranodal site	
0–1	744 (65.7%)
2–3	324 (28.6%)
4	32 (2.8%)
> 4	32 (2.8%)
IPI score	
High/high-intermediate	485 (42.8%)
Low/low-intermediate	647 (57.2%)
Missing	0 (0%)

Abbreviations: ABC, activated B-cell; BCL2, B-cell lymphoma 2; ECOG PS, Eastern Cooperative Oncology Group performance status; EORTC QLQ-C30, European Organization for Research and Treatment of Cancer Quality of Life Questionnaire; FACT-Lym LymS, Functional Assessment of Cancer Therapy-Lymphoma Lymphoma Subscale; GCB, germinal center B-cell; IPI, International Prognostic Index; LDH, lactate dehydrogenase; TMTV, total metabolic tumor volume

### Adjusted associations between changes of the PROM score and risks of PFS

**Fig. 1:**
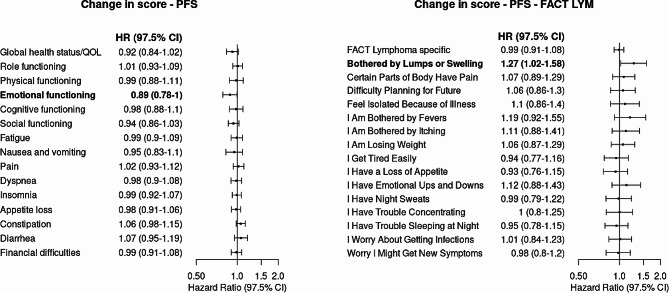
Risk of Progression-Free Survival When Measured by Change in EORTC QLQ-C30 Score and FACT-Lym LymS Score, Respectively, From Baseline to Cycle 3 Day 1 in the Study Sample (*N* = 1,132). Abbreviations: EORTC QLQ-C30, European Organization for Research and Treatment of Cancer Quality of Life Questionnaire; HR, hazard ratio; QOL, quality of life; FACT-Lym LymS, Functional Assessment of Cancer Therapy-Lymphoma Lymphoma Subscale

Among the 1,132 patients, 224 PFS events (i.e., disease progression or death) were observed, with progressive disease accounting for the majority (*n* = 138; 62%) of the events. Results from the multivariate analysis suggest that, after adjusting for confounders (i.e., disease risk at diagnosis, cell of origin, *BCL2* status, total metabolic tumor volume, and baseline PRO), when measured by EORTC QLQ-C30, every 10-point increase in emotional functioning from baseline to C3D1 was associated with an 11% lower risk of progression or death (HR, 0.89; 97.5% CI, 0.78 to 1; *P =* 0.02). When assessing the risk of PFS with HRQOL measured by FACT-Lym LymS, every 1-point increase (worsening) in lumps or swelling symptoms, from baseline to C3D1, was associated with a 27% higher risk of disease progression or death (HR, 1.27; 97.5% CI, 1.02 to 1.58; *P* = 0.01) **(**Fig. 1**).**

### Adjusted associations between changes of the PROM score and risks of OS

**Fig. 2:**
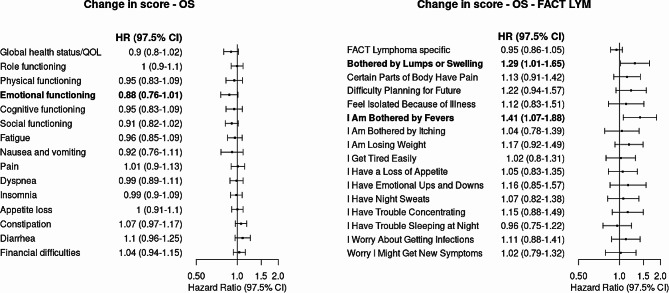
Risk of Overall Survival When Measured by Change in EORTC QLQ-C30 Score and FACT-Lym LymS Score From Baseline to Cycle 3 Day 1 in the Study Sample (*N* = 1,132). Abbreviations: EORTC QLQ-C30, European Organization for Research and Treatment of Cancer Quality of Life Questionnaire; HR, hazard ratio; QOL, quality of life; FACT-Lym LymS, Functional Assessment of Cancer Therapy-Lymphoma Lymphoma Subscale

For risk of OS, when measured by EORTC QLQ-C30, every 10-point increase in emotional functioning from baseline to C3D1 was associated with a 12% lower risk of death (HR, 0.88; 97.5% CI, 0.76 to 1.01; *P* = 0.04). When assessing risk of OS with HRQOL measured by FACT-Lym LymS, every 1-point increase in fever symptoms from baseline to C3D1 was associated with a 41% higher risk of death (HR, 1.41; 97.5% CI, 1.07 to 1.88; *P* = 0.01). Every 1-point increase in lumps or swelling symptoms from baseline to C3D1 was associated with a 29% higher risk of death (HR, 1.29; 97.5% CI, 1.01 to 1.65; *P* = 0.02). No significant associations were observed between changes in other symptoms such as itching (HR, 1.04; 97.5% CI, 0.78 to 1.39; *P* = 0.76) or weight loss (HR, 1.17; 97.5% CI, 0.92 to 1.49; *P* = 0.13) and risk of death (Fig. 2**)**.

## Discussion

To the best of our knowledge and a review of the literature, this is the first exploratory study looking at the impact of changes in PROs, especially lymphoma symptoms, over time on survival in DLBCL. In clinical practice, of the B-cell lymphoma symptoms, “B symptoms,” such as fever, are significant to the prognosis and staging of the disease. Other symptoms, such as itching and fatigue, do not have the same prognostic importance as B symptoms [14]. Consistent with this, our findings suggest that not all changes in all B-cell lymphoma symptoms hold the same prognostic value for DLBCL survival. Worsening in a patient’s perception of lumps or swelling was statistically significantly associated with an increased risk of disease progression and death. Worsening in a patient’s perception of fever was statistically significantly associated with an increased risk of death. No significant associations were observed between survival and changes in other symptoms, such as itching. A clearer understanding of patient-reported symptoms can improve the doctor-patient relationship and also help them make more informed treatment decisions. This study emphasizes the importance of understanding more about the changes in PROs, including cancer symptoms.

The diagnosis of cancer often results in increased emotional stress and anxiety for patients and their caregivers and families [15]. In our study, improvements in emotional functioning from baseline to C3D1, when measured by the EORTC QLQ-C30, were statistically significantly associated with improved PFS and OS. These improvements in our study patients could be due to factors such as greater optimism upon entering a clinical trial and receiving treatment, better social support, or an underlying improvement in physical functioning [15].

The focus of this study was on assessing changes in PROs over time and our results suggest that when adjusting for baseline scores, some PROs are important when assessed longitudinally. However, it is important to note that even baseline PRO scores alone have been shown to be prognostic of survival in DLBCL [4]. Similarly, some baseline PROs in our analysis, such as the physical functioning and global health status/QOL were prognostic of PFS and OS [Supplemental figures S1 and S2]. These results are consistent with those reported by Huang et al. [4]; however, changes in these PROMs were not statistically significantly associated with survival.

When designing studies, researchers should balance the need for information and patient burden. In order to obtain the most important data and minimize response burden, it is important to use instruments that include the most appropriate content [16]. Disease-specific instruments have been shown to be important at measuring change in patient status [17], and our study supports this. Changes in the lymphoma specific symptoms as measured by the FACT-Lym LymS were related to survival, whereas the treatment-related symptom scales included in the EORTC QLQ-C30 were not prognostic.

This analysis has some limitations. First, as this is an exploratory study, findings will need to be confirmed in future research. Second, the change analysis of individual LymS items is novel and no prior studies have investigated the MID of individual items from this scale. This will need to be further validated, ideally via the patient interviews, in future studies. Third, we do not know why changes in lymph swelling is a better prognostic marker (for both PFS and OS) than fever (for OS only). One possible explanation is that some of the fever worsening may have occurred during the treatment after disease progression. We will further investigate this in the future. Fourth, the data used for this study were collected from a clinical trial setting. Therefore, it may not fully represent the real-world routine practice for the larger DLBCL population. Fifth, it is also important to note that given that this was a hypothesis-generating study, multiple testing was not included in the analysis. Finally, due to the high mortality rate of DLBCL and the significant drop in patients available for PRO assessment in the GOYA trial after C3D1, this secondary analysis was limited to the period from baseline to C3D1. Future studies could examine long-term changes in PROs.

## Conclusions

Analyses from this study suggest the importance of changes in some PROs on survival in DLBCL. Worsening in some B-cell lymphoma symptoms, specifically lumps or swelling and fever, was associated with an increased risk of disease progression or death. No significant associations were observed between survival and changes in other symptoms, such as itching. Improvements in emotional functioning were also found to be associated with improved PFS and OS. Due to the exploratory nature of the analysis, findings of the current study need to be confirmed in future research.

### Electronic supplementary material

Below is the link to the electronic supplementary material.

Supplementary Material 1

Supplementary Material 2

## Data Availability

Qualified researchers may request access to individual patient level data through the clinical study data request platform (https://vivli.org/). Further details on Roche’s criteria for eligible studies are available here (https://vivli.org/members/ourmembers/). For further details on Roche’s Global Policy on the Sharing of Clinical Information and how to request access to related clinical study documents, see here (https://www.roche.com/research_and_development/who_we_are_how_we_work/clinical_trials/our_commitment_to_data_sharing.htm).

## Abbreviations

ABC: Activated B-cell
BCL2: B-cell lymphoma 2
ECOG PS: Eastern Cooperative Oncology Group performance status
C3D1: Cycle 3 Day 1
CHOP: Cyclophosphamide, doxorubicin hydrochloride [Adriamycin], vincristine sulfate [Oncovin], and prednisone
G-CHOP: Obinutuzumab (G) in combination with CHOP
R-CHOP: Rituximab (R) with CHOP
DLBCL: Diffuse large B-cell lymphoma
EORTC QLQ-C30: European Organization for Research and Treatment of Cancer Quality of Life Questionnaire
FACT-Lym LymS: Functional Assessment of Cancer Therapy-Lymphoma Lymphoma Subscale
GCB: Germinal center B-cell
HR: Hazard ratio
IPI: International Prognostic Index
LDH: Lactate dehydrogenase
MID: Minimal important difference
OS: Overall survival
PRO: Patient-reported outcome
PROM: Patient-reported outcomes measure
PFS: Progression-free survival
SD: Standard deviation
TMTV: Total metabolic tumor volume
