# Supplementary material for: Are changes in patient-reported outcomes prognostic for diffuse large B-Cell lymphoma survival? Results from the GOYA trial
Source: J Patient Rep Outcomes. 2024 Mar 18;8:31. doi: 10.1186/s41687-024-00708-w (PMC10948672; doi:10.1186/s41687-024-00708-w)
Supplement: Supplementary file 2 — Supplementary Material 2 [file 41687_2024_708_MOESM2_ESM.docx]

**Supplemental Figure S2. Hazard Ratios for Baseline PROs in Cox Regression Models to Assess Risk of Overall Survival (OS) (N=1,132)**

**
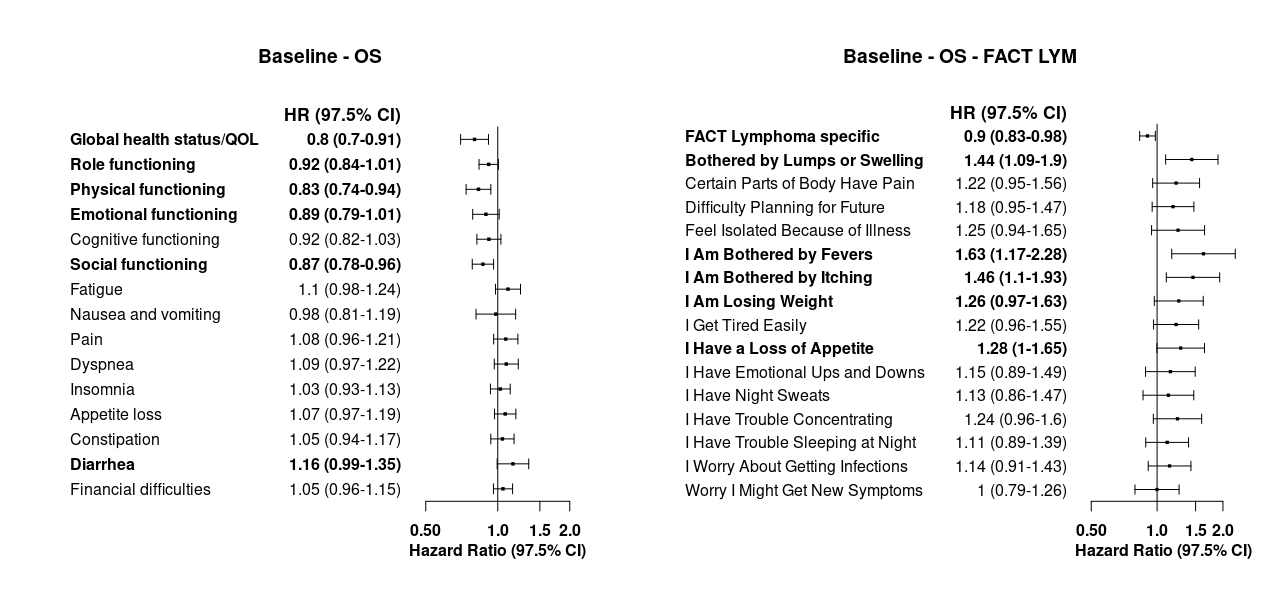
**

Abbreviations: EORTC QLQ-C30, European Organization for Research and Treatment of Cancer Quality of Life Questionnaire; HR, hazard ratio; QOL, quality of life**;**  FACT-Lym LymS, Functional Assessment of Cancer Therapy-Lymphoma Lymphoma Subscale
